# Effects of Short-Term Plyometric Training on Agility, Jump and Repeated Sprint Performance in Female Soccer Players

**DOI:** 10.3390/ijerph18052274

**Published:** 2021-02-25

**Authors:** Marcin Maciejczyk, Renata Błyszczuk, Aleksander Drwal, Beata Nowak, Marek Strzała

**Affiliations:** 1 Department of Physiology and Biochemistry, Faculty of Physical Education and Sport, University of Physical Education, 31-571 Kraków, Poland; 2 Department of Physical Education, Faculty of Health Sciences, University of Applied Sciences, 33-100 Tarnów, Poland; renatablyszczuk@interia.pl (R.B.); beatan72@gmail.com (B.N.); 3 Doctoral School, University of Physical Education, 31-571 Kraków, Poland; aleksanderdrwal@onet.pl; 4 Department of Water Sports, Faculty of Physical Education and Sport, University of Physical Education, 31-571 Kraków, Poland; marek.strzala@awf.krakow.pl

**Keywords:** physical fitness, training, performance, soccer, plyometric, female

## Abstract

The aim of the study was to determine the effects of short-term (4 weeks, twice a week: 8 sessions) plyometric training on agility, jump, and repeated sprint performance in female soccer players. The study comprised 17 females performing this sports discipline. The players were randomly divided into two groups: with plyometric training (PLY) and the control (CON). All players followed the same training program, but the PLY group also performed plyometric exercises. Tests used to evaluate physical performance were carried out immediately before and after PLY. After implementing the short PLY training, significant improvement in jump performance (squat jump: *p* = 0.04, ES = 0.48, countermovement jump: *p* = 0.009, ES = 0.42) and agility (*p* = 0.003, ES = 0.7) was noted in the PLY group. In the CON group, no significant (*p* > 0.05) changes in physical performance were observed. In contrast, PLY did not improve repeated sprint performance (*p* > 0.05) among female soccer players. In our research, it was shown that PLY can also be effective when performed for only 4 weeks instead of the 6–12 weeks typically applied.

## 1. Introduction

Men’s soccer is one of the most popular sports in the world. On the other hand, women’s soccer is a dynamically developing sport discipline, systematically rising in popularity. Compared to men’s soccer, relatively little research has been carried out on the effects of typical training methods applied in female soccer players [1]. Soccer is a sport discipline requiring comprehensive fitness preparation (aerobic and anaerobic) and motor skills. Although most of the efforts performed during the game are aerobic, anaerobic efforts are still crucial in decisive elements of the game, i.e., ones after which goals are scored.

Repeated sprints, reactive agility (change of direction speed), and jumps are the basic elements of motor and fitness preparation of players [2,3]. One of the most popular exercise methods, also used in soccer, is plyometric training (PLY) [4]. It is considered an important component of athletic strength as well as jump performance and injury prevention programs [5,6]. Plyometric training consists of bodyweight jumping-type exercises, using stretch-shortening cycle muscle action. This cycle enhances the ability of neural and musculotendinous systems to produce maximal force in the shortest possible time, prompting the implementation of plyometric exercise as a bridge between strength and speed [7]. PLY elicits a positive, small-to-moderate effect on jumping, sprint performance, and lower body muscle strength in healthy, recreationally active adults or athletes [8]. In competitive sport, this training, initially used to improve jump performance, has also proved effective in team sports (including soccer) by increasing agility (change of direction speed), sprint, and endurance performance [4,9,10,11,12]. However, these studies were mostly conducted among men [1]. In a previous study [11], it was suggested that adaptations to plyometric training do not differ between men and women, and the effectiveness of this training has also been confirmed in female soccer players. Following PLY, improvement was noted in speed, jump, kick, and aerobic endurance performance, without initiating changes in anaerobic performance [1,4,13].

Despite demonstrating the effectiveness of this training type in women, there is also a need for further research among soccer players of this sex [1,14,15]. This is due to the small number of studies (in comparison to the amount of research conducted in male soccer players) and the variety of study protocols used, which vary in duration, frequency, intensity, and volume. Typically, plyometric training is carried out over a period of several days or weeks (6–12 weeks), at a training frequency of 1–3 sessions per week, and a maximal to near-maximal intensity [4]. Ramirez-Campillo et al. [16] indicated that in future research, specific dose-response relationships following PLY must be identified. An interesting direction in this research is determining the minimum duration of PLY. Meta-analyses conducted to date indicate that longer training durations (10 weeks) provide greater improvement in jump performance [1]. However, it seems that shortening the duration of plyometric training, while remaining effective, may be crucial in developing training plans. The preparatory period in soccer is quite short compared to the duration of the entire season and the number of games played. At the time of preparation, coaches must holistically focus on all aspects of training, from endurance, strength, power, speed, and ending with jumping. During this period, time should also be devoted to tactical and technical training. The short time spent on conditioning and motor preparation requires the development of new training protocols to maximize the effects of training carried out over such a short duration.

Therefore, the aim of the study was to determine the effects of short-term (4 weeks, twice a week: 8 sessions) plyometric training on agility, jump, and repeated sprint performance in female soccer players. We hypothesized that a short plyometric training intervention would significantly improve agility, jump, and speed among these athletes.

## 2. Methods

### 2.1. Study Design

This research was designed as a parallel, randomized, controlled trial. The female athletes were randomly divided into 2 groups: plyometric training (PLY) and the control (CON). In groups corresponding to playing position (goalkeepers, defenders, midfielders, forwards), the players drew a group (CON, PLY) in which they then trained during the intervention. All players followed the same routine training program, except for the PLY group who additionally performed plyometric exercises after training properly (8 plyometric training sessions), twice a week (Monday and Friday), and for 4 weeks. The tests used to evaluate physical performance were performed immediately before and after the plyometric training (1 week prior and post its completion).

The study included 17 soccer players (1st league, Poland), who had no injuries at the beginning of the study. The inclusion criteria were: lack of experience in plyometric training (did not perform PLY in the last 24 months), and no injury in the 6 months preceding the study. Research was carried out during the preparation period for the spring round. Over the course of the project, 2 players were excluded from the study (injury not related to plyometric training and resignation). Ultimately, 7 players from the PLY group and 8 from the CON group obtained complete results. The examined players had no previous experience in plyometric training. Before and after the plyometric training intervention, height and weight measurements were taken for all the subjects. Furthermore, agility (Illinois Agility Test (IAT)), jumping performance (squat jump (SJ), countermovement jump (CMJ)), and repeated sprint ability (Running Anaerobic Sprint Test (RAST)) tests were performed.

Testing was carried out in a sports hall, always at the same time of the day. Jump tests (SJ, CMJ) were performed first, followed by IAT and RAST. The interval between subsequent tests was approximately 15 min. Before the day of the proper test, the players did not perform heavy training, and were instructed to rest as well as hydrate.

All participants were acquainted with the purpose and course of research. They also provided their written consent to participate in the project. In the case of soccer players under the age of 18, their parents submitted additional, written consent.

### 2.2. Participants

Female soccer players aged 16 to 26 participated in the study. The average age of the players in the PLY group was 21 ± 3 years, while in the CON group—18.2 ± 1.8 years. The training experience of the female players from both groups was similar and totaled: 9.75 ± 3.75 and 8.9 ± 2.5 years, respectively. During the observation period of 1 week, the players performed an average of 4.4 ± 1.7 training sessions and played 1 match. The mean body height of soccer players was: PLY 164.5 ± 6.91 cm, CON: 161.7 ± 4.3 cm; and body mass was 61.3 ± 13.86 kg and 55.0 ± 5.39 kg, respectively. At baseline, there were no significant differences between PLY and CON in: body height (f = 0.85, *p* = 0.37), body mass (f = 1.25, *p* = 0.26), training experience (f = 0.31, *p* = 0.58), or age (f = 4.52, *p* = 0.06).

### 2.3. Anthropometric Measurements

The body height of the female athletes was measured to the nearest 1 mm using a stadiometer (Seca, Hamburg, Germany). Body mass was determined to the nearest 0.1 kg with a scale (Tanita TBF-538, Tokio, Japan).

### 2.4. Squat and Countermovement Jumps

Jump height was assessed by the SJ and CMJ performed without arm swing (i.e., hands placed on hips). During the SJ, participants were instructed to start from a semi-squatted position and make no countermovement. In CMJ, they began from an erect position and made a downward movement before taking off from the floor. During the CMJ, there was no interval for rest between the 2 phases of the exercise (eccentric and concentric phases). The participants performed 3 trials for each mode, with the best result used for analysis. Jump height during the measurements was evaluated via the Optojump system (Microgate, Bolzano, Italy).

### 2.5. Illinois Agility Test

The IAT was administered using a version standardized on the basis of previous papers [17,18,19]. The participant began the test lying prone on the floor, behind the starting line, with arms along her side and head turned to the side or facing forwards. On command, the athlete sprinted to finish the agility course. Performance was recorded using an electronic timing system (Witty, Microgate, Bolzano, Italy). The photocells were positioned at the starting and finish lines at a height of approximately 1.00 m. The best performance of the 2 trials was recorded for statistical analyses.

### 2.6. Running Anaerobic Sprint Test

The RAST was applied by the participants performing 6, 35-m maximal sprints, with a 10-s interval between each trial. The time for each run was measured using 2 photocells (Witty, Microgate, Italy). The power (P) of each sprint was then calculated using the formula: Power = (Body Mass × Distance^2^)/Time^3^ [W], and the best result was considered as maximal power (Pmax). Pmax was presented as an absolute value (in Watts), relative to body mass (W/kg). Mean power (Pmean) was considered the average power from 6 runs. Fatigue index (FI) was calculated using the formula: FI = (Pmax–Pmin) ÷ Σt6 [W/s] [20,21,22].

### 2.7. Plyometric Training

Both groups carried out the same training intervention planned by the coach. However, the players from the PLY group, after training properly, performed additional plyometric exercises twice a week, which were specifically prepared for the needs of this experiment. It was the only difference in training between the groups under study. The training protocol lasted 4 weeks and was performed in a sports hall. The volume of training—determined by the number of repetitions, i.e., “contacts” with the ground—systematically increased in the first 3 weeks and was then decreased in the final week to a level similar to the second one. In the first week, during the plyometric training session, the players performed a total of 107 contacts, in the second—133, in the third—159, and 125 contacts in the last week (Table 1). The total number of jumps was 524 over a 4-week period. The time between each set was 2 min, and subsequent repetitions were performed immediately after completing the previous one. In the training protocol, 18 different exercises were used. Therefore, the training was not of strict exercise type (e.g., only vertical/horizontal/diagonal, cyclic/acyclic or unilateral/bilateral exercises), but a combination of different plyometric efforts. Exercise during weeks 1–3 was different, while during weeks 2 and 4, it was the same. Female athletes were encouraged to exercise at or near to maximal intensity. A detailed description of the exercises is presented in Table 1, and their graphic presentation may be found in the attached supplement (Figure S1).

### 2.8. Statistical Analysis

The research results are presented as means and standard deviations (mean ± SD). In order to assess the significance of differences between groups, before and after the training intervention, analysis of variance (ANOVA) with repeated measurements was used. Following, post hoc analysis was carried out using Tukey’s test. Data distribution was checked using the Shapiro–Wilk test. Homogeneity of variance within the groups was tested via Levene’s test (variance of the analyzed parameters was similar in both groups). The effect size (ES: Cohen’s d) was also calculated and interpreted as small (0.20), medium (0.50), or large (0.80) [23]. Statistical analysis of the results was performed using Statistica 12.0 software (StatSoft, Tulsa, OK, USA). The differences in all analyzed indices were considered statistically significant at the level of *p* < 0.05.

## 3. Results

In the study, significant changes were noted for IAT (f = 15.77, *p* = 0.002), SJ (f = 9.06, *p* = 0.01), and CMJ (f = 11.15, *p* = 0.005) under the influence of training. Post hoc analysis allowed to demonstrate that significant changes in these indices were only observed in the PLY group, while in CON, they were not considered significant (Table 2). There were no effects of plyometric training on the level of average and maximal power, or the fatigue index measured in RAST (Pmean: f = 2.77, *p* = 0.12; Pmax: f = 2.26, *p* = 0.15; FI: f = 0.05, *p* = 0.83).

The studied groups did not differ with regard to the examined indices (Pmax: f = 1.52, *p* = 0.23; Pmean: f = 0.86, *p* = 0.37; FI: f = 3.10, *p* = 0.10; IAT: f = 0.62, *p* = 0.45; SJ: f-0.14, *p* = 0.90; CMJ: f = 0.07, *p* = 0.78). Moreover, there were no significant interactions between the analyzed factors (group x training) (Pmax: f = 0.23, *p* = 0.63; Pmean: f = 0.06, *p* = 0.81; FI: f = 0.07, *p* = 0.80; IAT: f = 4.29, *p* = 0.06; SJ: f = 1.33, *p* = 0.27; CMJ: f = 3.59, *p* = 0.08). In the examined groups, body mass did not undergo change between the trials (PLY: *p* = 0.88; CON: *p* = 0.12).

## 4. Discussion

The results of our study indicate that even a short plyometric training intervention can be effective in improving the physical fitness of female soccer players. After applying this type of training, we noted significant improvement in jump performance (SJ, CMJ) and agility (IAT). However, this training intervention did not improve repeated sprint performance. Despite the much shorter plyometric training period (4 weeks vs. 6–12 weeks, as recommended in previous studies), we obtained similar training effects as those noted in previous reports. We did not observe significant differences between groups or significant interactions between main factors (group and training). Lack of interaction suggests that in both groups, changes of a given variable during training were similar, but in the PLY group, the change was more dynamic (and statistically significant). The CON group also performed routine soccer training, which could have also improved the tested abilities. In the CON group, there were non-significant changes in some of the parameters, but adding plyometric training to the routine in the PLY group caused significant beneficial changes in some of the measured parameters.

Our research results are consistent with the data presented in the meta-analysis by Sanchez et al. [4], who showed that PLY is effective in soccer players to improve SJ, CMJ, and agility, but not repeated sprint ability. However, the novelty of this study is that we demonstrated the same beneficial effects of PLY after only four weeks of the intervention. To our best knowledge, this is the first study in which the effectiveness of such short training sessions has been noted in female soccer players. Similar short-term training [24,25] among soccer players lasted 7–8 weeks, but it should be emphasized that the soccer players participating in our study had no previous experience with plyometric training, despite several years of training, which may have had an impact on the size of the observed changes. The recruitment of players for the study was intentional—our assumption was that greater improvement in physical fitness will be more likely to occur in players not adapted to plyometric training. Of course, in the training of these players so far, jumping exercises were performed, but not plyometric training. Therefore, it is difficult to determine whether similar effects would have been observed in female players having some previous experience with plyometric training.

In previous studies on soccer players, RAST had rarely been used to assess anaerobic performance. This test provides a lot of useful data in assessing anaerobic fitness. This test allows not only to evaluate an athlete’s speed at a distance of 35 m, but also makes it possible to calculate Pmax from sprints, Pmean from 6 sprints, and fatigue index—as well as anaerobic endurance and repeated sprint ability. There were no significant changes in maximal and average power, or FI measured in RAST under the influence of PLY in the studied soccer players. Thus, plyometric training proved ineffective in improving repeated sprint performance. The lack of changes in fatigue index (anaerobic endurance) recorded in our study may result from the fact that the examined players, demonstrated very good anaerobic endurance before the beginning of PLY exercise—the FI in both groups was only 2–3 W/s.

A possible explanation for the effectiveness of short-term plyometric training should be seen in the characteristics of this training type, which was performed at the recommended weekly frequency (1–3 per week). Therefore, it did not differ from that applied in previous research [4]. However, in some studies, it is indicated that training at a lesser frequency may be more effective, nonetheless—in the study by Yanci et al. [26], it was shown that a 1-day per week plyometric training program can be more efficient than a 2-day per week intervention with regard to improving futsal players’ physical performance. Rubley et al. [13] indicated that once-weekly, low-impact plyometric training programs may be effective for improvement in vertical jump and kicking distance among female soccer players. When training several times a week, the minimum inter-session rest should be 48 h, which is an adequate, minimal recovery time [4].

The total number of jumps in our study was 524 over a 4-week period, with a weekly volume of 107–159. In previous studies, training volume was lower: 70–140 jumps per week [27], or: 90–220 jumps per week in the study by Ozbar et al. [28]. Typically, the total amount of jumps ranges between 800 to 3240 jumps in 6–12 weeks [4]. Thus, it may be concluded that the applied volume and frequency of training were similar to those implemented in previous studies. In earlier research, it was demonstrated that a higher PLY frequency has no additional effects on female soccer players’ physical fitness [27,29]. A moderate PLY volume is recommended for inexperienced female athletes [4]. Moreover, a moderate volume may be as effective as a program with a greater volume [30].

The interval duration for rest between sets and repetitions used in our study was similar to those reported in previous studies. Usually, rest between sets ranges from 30 s up to 300 s, and for inter-repetition rest, values from 5 up to 60 s have been indicated (Sanchez, 2020). For optimal and effective recovery, 30–120 s of inter-set rest, and 15 s of inter-repetition rest, are recommended for soccer players [31,32].

The exercises used in our plyometric training intervention were varied and totaled as many as 18, implemented in various combinations. The training can be classified as combined PLY with another type of drill. It was performed in various planes (vertical, horizontal, diagonal), and both unilaterally and bilaterally. In our opinion, it may be this training form that caused our short-term intervention to be as effective as longer training sessions, bearing in mind that the frequency, volume, as well as inter-session time were comparable to those suggested in previous studies. The trials by Ramirez-Campillo et al. [33,34] indicated that the combination of unilateral/bilateral and vertical/horizontal drills were more advantageous in inducing superior performance improvement in young soccer players. However, this requires confirmation by carrying out further research among female soccer players, in which different forms of plyometric training would be carried out and compared over a short period of time.

## 5. Limitation of the Study

Limitations of the study are the small number of participants and the results are restricted to inexperienced players. Further research should be carried out to verify the effectiveness of short-term plyometric training among athletes already experienced in this type of training.

## 6. Conclusions

The results of our research are of great applicative significance. Currently, research is being carried out to optimize plyometric training in terms of its duration, volume, frequency and characteristics of the implemented exercises in order to reach the greatest possible effectiveness of this type of training. After implementing the short PLY training, significant improvement in jump performance and agility was noted in the PLY group. In the CON group, no significant changes in physical performance were observed. In contrast, PLY did not improve anaerobic performance among female soccer players. In our research, it was shown that PLY can also be effective when performed for only 4 weeks instead of the typically applied 6–12 weeks. Due to the usually short pre-season and long proper season in soccer, coaches can prepare their training plan better and more effectively. Such a short plyometric training procedure can also be successfully used during the several-week-long break between the autumn and spring rounds.

## Figures and Tables

**Table 1:** Characteristics of the applied training intervention.

Weeks	Day	Volume	Number of Exercise	Exercise Description	Sets × Reps	Intensity (Volume—No. of Contacts)
I	MON	50	1	Double-leg jump over 5 hurdles	4 × 5	low (107)
			2	Single-leg jump over 5 hurdles	3 × 5	
			3	Forward double-leg jump 1 hurdle and sideways single-leg jump over 1 hurdle, and sideways to target and back	3 × 5	
	FRI	57	4	Double-leg jump over 5 hurdles and single-leg jump sideways to target at the end	4 × 6	
			5	Single-leg jump over 5 hurdles and double-leg jump over 1 hurdle sideways at the end	3 × 6	
			6	Forward double-leg jump over 1 hurdle and single-leg jump sideways over 1 hurdle and sideways to target and back with 90° turn at the end	3 × 5	
II	MON	68	7	3 single-leg jumps to target and sideways, single-leg jump between hurdle 4x, single- leg jump over hurdle at the end	2 × 12	Moderate (133)
			8	Single-leg jump over 3 hurdles with sideways jump to target 2×, single-leg sideways jump over 1 hurdle at the end	2 × 10	
			9	Double-leg jump over 1 hurdle and single-leg jump to designated area and return, repeated for other side and return	3 × 8	
	FRI	65	10	Single-leg jump over 1 hurdle and sideways jump over 1 hurdle, 3 single-leg jumps to target and sideways single-leg jump between hurdle 3×, forward single-leg jump over hurdle at the end	2 × 10	
			11	Single-leg forward jump over 3 hurdles, halfway, double-leg sideways jump over 1 hurdle and single-leg forward jump over 3 hurdles	3 × 7	
			12	Double-leg jump over 1 hurdle and single-leg jump to designated area and return, the same for other side and return	3 × 8	
III	MON	95	13	Double-leg jump over 1 hurdle, single-leg jump to designated area 2× and on the other side, double-leg forward jump over 1 hurdle 2×, at the end, double-leg jump over 1 hurdle and 2 single-leg jumps to target	3 × 15	High (159)
			14	Single-leg forward jump over 3 hurdles, double-leg jump with 90° turn over 1 hurdle, 2 single-leg forward jumps 1× and double-leg forward jump with 90° turn, 2 single-leg jumps with 90° final turn	3 × 10	
			15	Double-leg forward jump 1×, single-leg jump to designated areas, 3x sideways jump over hurdle and 3× jump to target, double-leg forward jump 1× and 2 single-leg jumps	2 × 10	
	FRI	64	16	Single-leg jump over 1 hurdle, sideways jump to designated area 2x, at the end, 1 single-leg jump forwards with 1 sideways jump	2 × 10	
			17	3 forward single-leg jumps, 1 double-leg forward jump with 90^0^ turn, 3 single-leg forward jumps, 2 double-leg jumps over hurdle with 90^0^ turn at the end	2 × 10	
			18	Sideways single-leg jump from designated areas above hurdles	4 × 6	
IV	MON	68	7	3 single-leg jumps to target and sideways single-leg jump between hurdle 4×, single-leg jump over hurdle at the end	2 × 12	
			8	Single-leg jump over 3 hurdles with sideways jump to target 2×, single-leg jump over 1 hurdle at the end	2 × 10	Moderate (125)
			9	Double-leg jump over 1 hurdle, single-leg jump to designated area and return, the same for other side, and return	3 × 8	
	FRI	60	10	Single-leg jump over 1 hurdle and sideways jump over 1 hurdle, 3 single-leg jumps to target and sideways one-single-leg jump between hurdle 3×, single-leg forward jump over hurdle at the end	2 × 10	
			11	Single-leg forward jump over 3 hurdles, halfway, double-leg sideways jump over 1 hurdle and single-leg forward jump over 3 hurdles	3 × 7	
			12	Double-leg jump over 1 hurdle and single-leg jump to designated area and return, the same for other side and return	2 × 8	

**Table 2:** Effects of plyometric training on agility, jump, and anaerobic performance in female soccer players.

Variable	Group	Training		p (post-hoc)	ES (Cohen’s d)
		Baseline	Post-Training		
Pmax/BM (W/kg)	PLY	6.23 ± 1.12	6.76 ± 1.42	0.60	0.42
	CON	6.29 ± 0.86	6.51 ± 1.33	0.96	0.20
Pmax (W)	PLY	370.8 ± 54.36	404.2 ± 65.71	0.48	0.56
	CON	344.8 ± 48.48	361.8 ± 78.31	0.98	0.27
Pmean (W)	PLY	314.96 ± 46.10	340.5 ± 74.43	0.52	0.42
	CON	294.79 ± 38.78	313.8 ± 51.94	0.76	0.42
FI (W/s)	PLY	3.0 ± 0.84	3.02 ± 0.60	0.99	0.03
	CON	2.58 ± 1.03	2.38 ± 1.42	0.98	0.16
IAT (s)	PLY	16.8 ± 0.88	16.2 ± 0.84	0.003	0.7
	CON	16.91 ± 0.58	16.72 ± 0.86	0.57	0.26
SJ (cm)	PLY	26.23 ± 5.14	28.63 ± 4.76	0.04	0.48
	CON	27.17 ± 4.38	28.24 ± 4.20	0.59	0.25
CMJ (cm)	PLY	28.11 ± 4.56	29.93 ± 5.01	0.009	0.42
	CON	28.14 ± 3.91	28.64 ± 3.91	0.75	0.13

Pmax: maximal anaerobic power from Running Anaerobic Sprint Test; BM—body mass; Pmean: mean anaerobic power from Running Anaerobic Sprint Test; FI: fatigue index from Running Anaerobic Sprint Test; IAT: Illinois Agility Test; SJ: squat jump, CMJ: countermovement jump; ES: effect size.

## Data Availability

Not applicable.

## Supplementary Materials

The following is available online at https://www.mdpi.com/1660-4601/18/5/2274/s1, Figure S1: Graphic scheme of exercises applied in the plyometric training of female soccer players.
