# Supplementary material for: Effects of Short-Term Plyometric Training on Agility, Jump and Repeated Sprint Performance in Female Soccer Players
Source: Int J Environ Res Public Health. 2021 Feb 25;18(5):2274. doi: 10.3390/ijerph18052274 (PMC7956435; doi:10.3390/ijerph18052274)
Supplement: Supplementary file 1 [file ijerph-18-02274-s001.pdf]

Figure S1. Graphic scheme of exercises applied in the plyometric training of female soccer players

1.

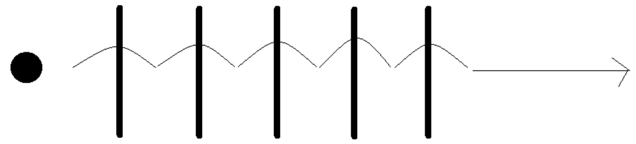

2.

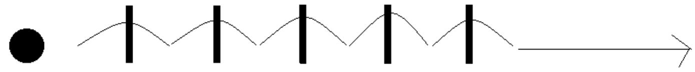

3. **4.**

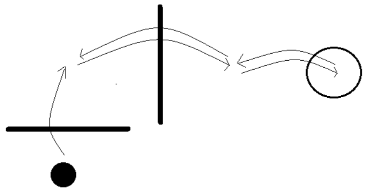

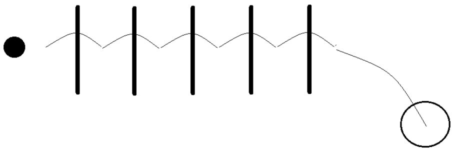

5. **6.**

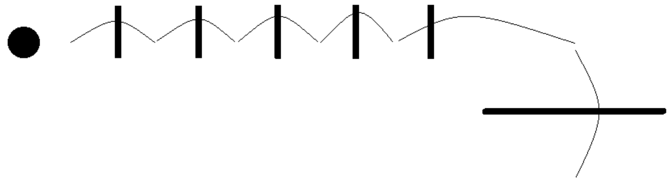

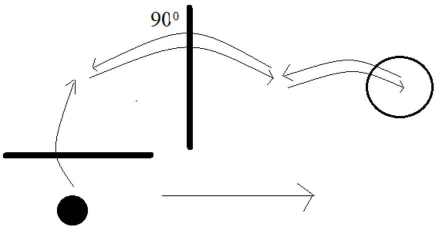

7. **8.**

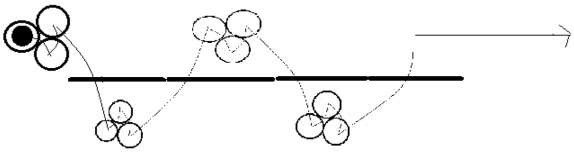

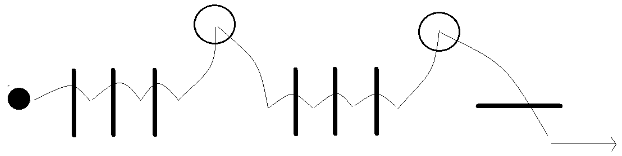

9. **10.**

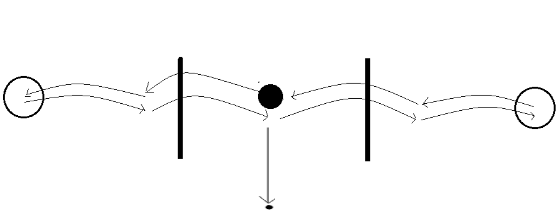

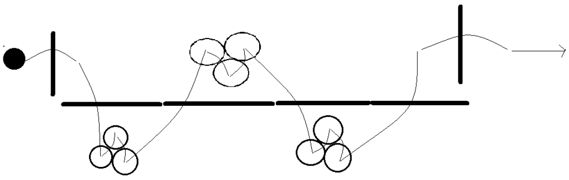

11. **12.**

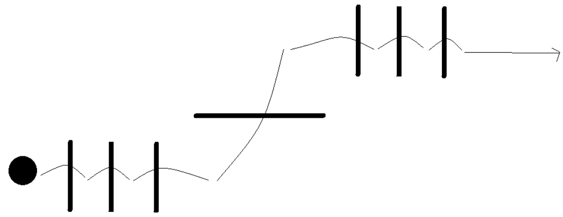

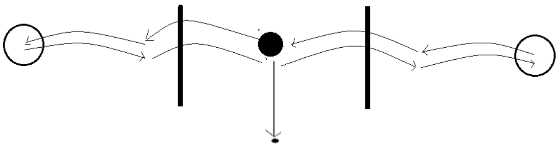

13. **14.**

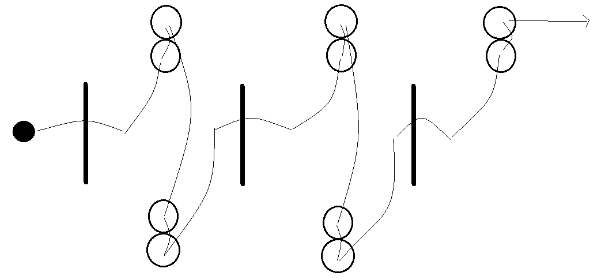

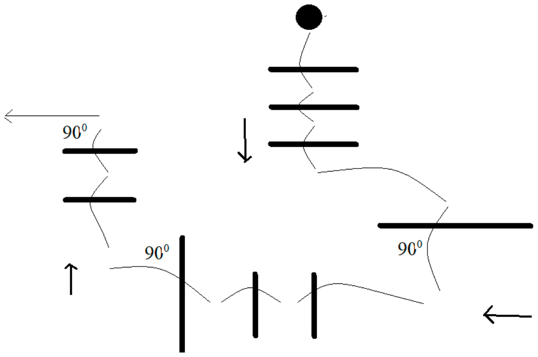

15.

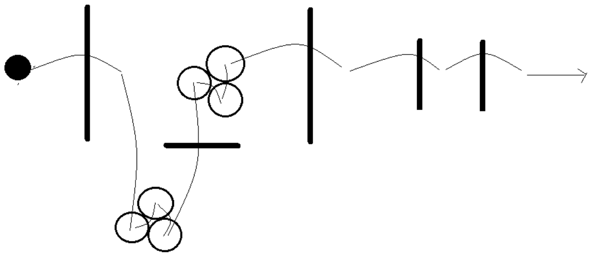

16.

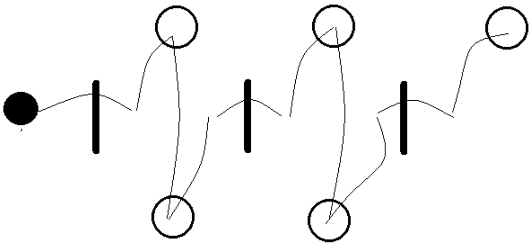

17.

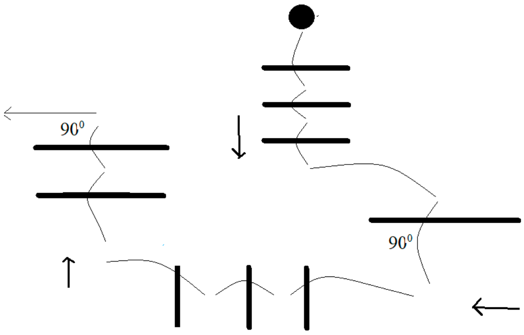

18.

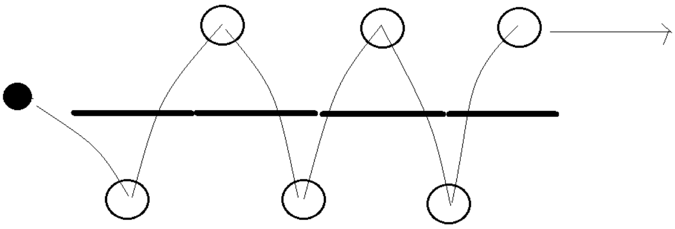

Legend

- person performing exercise

- direction

- gymnastics ring

1.

- hurdle (45 cm)

2.

- hurdle (10 cm)

3. / 4. / 5. / 6.

- hurdle (45 cm)
- hurdle (10 cm)

7. / 8.

- hurdle (10 cm)

9.

- hurdle (45 cm)

10.

- hurdle (10 cm)

11.

- hurdle (10 cm)

- hurdle (45 cm)

12.

- hurdle (45 cm)

13.

- hurdle (45 cm)

14.

- hurdle (10 cm)
- hurdle (45 cm)

15.

- hurdle (45 cm)
- hurdle (10 cm)

16.

- hurdle (10 cm)

17.

- hurdle (10 cm)
- hurdle (68 cm)

18.

- hurdle (10 cm)
